# Pragmatic Abilities in Children with Congenital Visual Impairment: An Exploration of Non-literal Language and Advanced Theory of Mind Understanding

**DOI:** 10.1007/s10803-012-1500-5

**Published:** 2012-03-22

**Authors:** Judith Pijnacker, Mathijs P. J. Vervloed, Bert Steenbergen

**Affiliations:** Behavioural Science Institute, Radboud University Nijmegen, P.O. Box 9104, 6500 HE Nijmegen, The Netherlands

**Keywords:** Visual impairment, Children, Pragmatic language, Advanced theory of mind, Non-literal stories

## Abstract

Children with congenital visual impairment have been reported to be delayed in theory of mind development. So far, research focused on first-order theory of mind, and included mainly blind children, whereas the majority of visually impaired children is not totally blind. The present study set out to explore whether children with a broader range of congenital visual impairments have a delay in more advanced theory of mind understanding, in particular second-order theory of mind (i.e. awareness that other people have beliefs about beliefs) and non-literal language (e.g. irony or figure of speech). Twenty-four children with congenital visual impairment and 24 typically developing sighted children aged between 6 and 13 were included. All children were presented with a series of stories involving understanding of theory of mind and non-literal language. When compared with sighted children of similar age and verbal intelligence, performance of children with congenital visual impairment on advanced theory of mind and non-literal stories was alike. The ability to understand the motivations behind non-literal language was associated with age, verbal intelligence and theory of mind skills, but was not associated with visual ability.

## Introduction

Some children with congenital visual impairment have a delay in theory of mind development (Brambring and Asbrock 2010; Green et al. 2004; McAlpine and Moore 1995; Minter et al. 1998; Peterson et al. 2000). Theory of mind refers to the ability to attribute mental states like intentions, beliefs, and desires to oneself and other people as a means to interpret and predict behavior. Theory of mind understanding is usually investigated by first-order false belief tasks, which examine whether a child can reason about the way in which people will act when holding a mistaken belief. For example, in the standard Sally-Ann task, a doll Sally puts a marble in her basket (Baron-Cohen et al. 1985). While Sally is away, another doll called Ann takes out Sally’s marble and puts it into her box. When Sally returns, she wants to play with her marble. The child is then asked where Sally will look for her marble. The correct answer is that Sally will look in her basket, but young children often report their own belief that Sally will look into the box, and thus failing to take into account that Sally has a false belief about the location of the marble. Typically, successful performance on first-order false belief tasks begins to emerge in children aged between 3 and 5 years (Wellman and Liu 2004; Wellman et al. 2001).

In children with congenital blindness or profound visual impairment, several studies have found a developmental delay in first-order false belief performance compared to typically developing children. However, the number of visually impaired children who are delayed and the age of theory of mind acquisition differed widely across studies, varying from age 7 to 12 years (Brambring and Asbrock 2010; Green et al. 2004; McAlpine and Moore 1995; Minter et al. 1998; Peterson et al. 2000; Roch-Levecq 2006). Brambring and Asbrock (2010) argued that the delayed onset of false belief performance might be due to visual components associated with previous false belief tasks. To overcome this potential confounding effect of visual components they constructed alternative false belief tasks where the role of vision is minimal. Using these alternative tasks Brambring and Asbrock (2010) found a delay in theory of mind development of at most 2 years. Specifically, by the age of 7, congenitally blind children were able to solve the alternative false belief tasks.

Delays in theory of mind development in visually impaired children have been attributed to a lack of access to visual information during interactions, like joint attention, mutual gaze, face expressions, and gestures (Green et al. 2004; Peterson et al. 2000; McAlpine and Moore 1995; Tadić et al. 2010; Pring et al. 1998). This kind of visual information is important for social communication as well. In line with this, children with congenital visual impairment have been shown to have difficulties with the use of language for pragmatic and social purposes, while structural language (e.g. articulation, grammar, vocabulary) was good or even superior (James and Stojanovik 2007; Tadić et al. 2010). This pattern of delay in socio-cognitive and socio-communicative development found in children with congenital visual impairment is also a core feature of sighted children with autism, and it has been suggested that children with congenital visual impairment are at risk of developing autism (Brown et al. 1997; Hobson and Bishop 2003; Hobson et al. 1999; Parr et al. 2010).

So far, research has focused on first-order theory of mind in visually impaired children, and most studies included only blind children, whereas the majority of visually impaired children is not totally blind. The present study set out to explore whether children with a broader range of congenital visual impairments have a delay in more advanced theory of mind understanding.

Advanced theory of mind can be examined by second-order false belief tasks. Second-order belief concerns the awareness that other people have beliefs about beliefs (e.g. Peter thinks that Ann thinks that the cake is in the drawer), whereas first-order belief refers to the awareness that other people have beliefs about a situation (e.g. Ann thinks that the cake is in the drawer). The ability to attribute second-order beliefs is considered to be required for more complex forms of social interaction and communication (Miller 2009; Filippova and Astington 2008; Caillies and Le Sourn-Bissaoui 2008; Perner and Wimmer 1985). The understanding of second-order mental states develops at a later age than first-order belief, usually between the ages 5 and 8 years (Miller 2009).

Another method to investigate advanced theory of mind is to examine non-literal language understanding. Non-literal language can be considered as part of pragmatics—that is, the use of language in communication—and encompasses, for instance, jokes, irony, sarcasm, figures of speech, lies and white lies. In non-literal language, the motivations behind an utterance diverge from the literal content. For example, someone saying that he loves the birthday present he received, may really love the birthday present or may be just polite and spare the giver’s feelings. Comprehension of the underlying intention of a non-literal utterance requires the attribution of mental states to another, and hence can be considered as a subtler test of theory of mind understanding (Happé 1994). Several studies have shown that both children and adults with autism have difficulty with identifying accurately the motivation behind a character’s non-literal utterance in everyday stories (Happé 1994; Kaland et al. 2008, 2002; Brent et al. 2004; Jolliffe and Baron-Cohen 1999; White et al. 2009), and that performance on theory of mind tasks is associated with non-literal language comprehension (Happé 1993; White et al. 2009). Moreover, one study demonstrated that children with visual impairment, like children and adults with autism, provided fewer context-appropriate mental state explanations for non-literal utterances than sighted controls (Pring et al. 1998). They suggested that there is a subtle difference between visually impaired children and sighted children in the use of language that requires insight into underlying intentions.

To date, little research has been dedicated to sophisticated theory of mind reasoning in children with congenital visual impairments (Pring et al. 1998; Roch-Levecq 2006). Additionally, as outlined above, most research has focused on blind children, whereas the majority of visually impaired children is not totally blind. In order to warrant ecological validity, it is important to investigate whether children with varying congenital visual impairments experience problems with (advanced) theory of mind reasoning. Moreover, it is important to determine whether there is a delay in pragmatic development in young as well as older children with visual impairments, as older children might catch up a possible delay. In brief, the central research question of the present study was: Do children with a broad range of congenital visual impairments—young children as well as older children—show a delay in advanced theory of mind development? To provide an answer to this question, we examined how visually impaired children (aged 6–13 years) with varying congenital visual impairments perform on (advanced) theory of mind reasoning and non-literal language comprehension compared to control children. Moreover, we examined whether task performance was related to visual acuity within the visually impaired group, as the extent of visual input (i.e. amount of vision) may play a mediating role. The purpose of this study was to provide a better insight into the relation between visual input and pragmatic development, that is, understanding of theory of mind and non-literal language.

## Methods

### Participants

Initially, a total group of 54 children between 6 and 13 years of age participated in this study. There were 30 children with a congenital visual impairment (VI), who were recruited from 4 special education schools for visually impaired children. Six children with a visual impairment were excluded from data analysis. One child was excluded because he received a diagnosis of autism spectrum disorder during the study, and another child was excluded because he was not testable due to attention problems. The other 4 children were excluded because their visual acuity was above 0.3 Snellen decimals. Hence, the VI group included 24 children. The control group consisted of 24 typically developing sighted children, who were recruited from local schools. All children had Dutch as their native language. Children with a diagnosis of autism spectrum disorder, cerebral visual impairment, severe hearing impairment, intellectual disability, or physical impairment were excluded. The groups were matched on gender, age, and verbal IQ. Participant characteristics are shown in Table 1. See “Appendix A” for individual diagnoses and visual acuities.

**Table 1:** Participant characteristics

	VI group (*n* = 24)	Control group (*n* = 24)	Group comparison	
Gender (M:F)	(11:13)	(11:13)		
Age (years, months)				
Mean (SD)	10.2 (2.0)	9.6 (1.4)	*t*(46) = −1.34	*p* > .10
Range	6.7–13.2	6.3–11.10		
Verbal IQ (WISC-III)				
Mean (SD)	101 (12)	107 (16)	*t*(46) = 1.74	*p* = .089
Range	78–129	75–136		
Visual acuity^a^				
Mean (SD)	0.11 (0.09)	n.a.		
Range	0–0.3	n.a.		

^a^Visual acuity of the best eye in Snellen decimals

*n.a.* not available

## Materials

### Theory of Mind

Theory of mind understanding was tested by different tasks, including two unexpected contents tasks, two first-order false belief stories, and two second-order false belief stories. See “Appendix B” for the complete stories and questions.

For the unexpected content tasks, each child was presented with an egg carton and asked what they thought what was in the egg carton (Peterson et al. 2000; Brambring and Asbrock 2010). After the child’s response, the egg carton was opened to reveal a set of bouncy balls. Next, the egg carton was closed and the child was asked two questions. First, what the child thought what was in the egg carton before it was opened (own false belief) and what was really in there (control question). Second, what a friend of the child would think what is in the egg carton (other false belief). In the other unexpected content task, the child was shown six wooden boxes that were covered (Brambring and Asbrock 2010). The boxes contained the following objects in sequence: lego cube—tea spoon—lego cube—tea spoon—lego cube—clothes peg. After box 3, the child was asked for each box what he would expect to find in the next box. In the final box, the content is unexpected on the basis of the previous content. After the unexpected content was revealed, the child was asked what he thought what was in the final box before he had opened it (own false belief), and what was really in there (control question). Second, the child was asked what a friend of the child would think what is in the final box (other false belief) if he would do the same task. In both unexpected content tasks, the objects could be recognized by touch, and hence the role of vision was minimal. The child was credited 1 point for a correct own false belief, and 1 point for a correct other false belief. The maximum total score for these two tasks was 4 points.

The first-order false belief stories were based on the classical Sally-Ann task (Baron-Cohen et al. 1985). The children listened to two stories where an object or person changed location in the absence of another person. The crucial question was where this person will look for the object or person. The child received 1 point for a correct answer and 1 point if the child could provide a right explanation why the person would think that the object or person was at that location. The maximum total score for these two tasks was 4 points.

The two second-order false belief stories involved a change of location of an object or a person. Two story’s characters have witnessed this change of location, but they are ignorant of each other’s knowledge (Astington et al. 2002; Perner and Wimmer 1985). Here the child was given 1 point for a correct answer, and 2 points for a justification in terms of mental states (e.g. “Because she does not know that Joris met the ice-cream man”) and 1 point for an explanation without referring to mental states (e.g. “The ice-cream man was in the park before”). The maximum total score for the second-order false belief stories was 6 points.

In all tasks, children were asked control questions to ensure that they understood the task or story. Because a correct answer on the false belief question, but no correct answer on the control questions, could be due to guessing, such responses were excluded from analysis and treated as missing values. In total, there were 6 missing values at the second-order false belief task (3 in VI group, 3 in control group).

### Non-literal Stories

The non-literal language task comprised 10 short stories in Dutch about everyday situations (see “Appendix C” for examples). The stories were based on the Strange Stories by Happé (1994), but adapted to typical Dutch situations, and constructed so that they did not appeal solely to visual experience. There were five types of stories, and two examples of each story type, including lies, white lies, jokes, figure of speech, and irony or sarcasm. The non-literal stories were followed by two questions. The first question had the form “Is it true what X said? Did X really Y?” to check comprehension. The second question involved a justification and had the form “Why did X say that?” A child could receive 1 point for the first question, as the first step in understanding non-literal language, is recognizing the non-literal content. For the second question, a child could receive 2 points for a fully correct explanation, 1 point for a partially correct explanation, and 0 points for an incorrect explanation (O’Hare et al. 2009). For instance, in one of the lie stories, the child received 2 points for a fully correct mental explanation (“He is afraid that his mother becomes angry at him”), 1 point for a partially correct mental explanation (“He does not like that the vase has been broken”), and 0 points if he gave a physical explanation (“The vase has been broken”), or a wrong mental state explanation (“He is joking”). In case of the jokes, the utterances could also be interpreted as a simile (e.g. “The dog is big like an elephant”; “The kittens are hairy like balls of wool”), and therefore such responses were credited with 1 point. The maximum score for each story type was 6 points, and for the complete set of stories 30 points. Finally, a control story was added that assessed the ability to make a causal inference.

### Children’s Communication Checklist (CCC-2 NL)

The Dutch version of Children’s Communication Checklist-2 (CCC-2 NL) was used to evaluate language and communication skills (Geurts 2007; Bishop 2003), as they may be important for theory of mind performance and non-literal language understanding. The CCC-2 is a standardized questionnaire for children from 4 to 15 years of age, and is completed by parents. The checklist contains 70 items that are grouped into 10 scales: (A) speech, (B) syntax, (C) semantics, (D) coherence, (E) inappropriate initiation, (F) stereotyped language, (G) use of context, (H) non-verbal communication, (I) social relations and (J) interests. Scores on individual subscales are converted to age-scaled standard scores with mean of 10 and SD of 3, based on a norm group of 2,580 Dutch children between 4 and 16 years of age.

For this study, we computed two composite scores: the General Communication Score (sum of standard scores on scales A to H) and the Pragmatics Score (sum of standard scores on scales E to H). Higher scores on the CCC-2 subscales and composite scores indicate more difficulties.

## Procedure

Informed consent from the children’s parents was obtained prior to participation in the study. Each child was tested individually in a quiet room at school. For children from whom verbal intelligence scores were not available, verbal intelligence was measured using the Wechsler Intelligence Scales for Children-III-NL (Kort et al. 2005). The CCC-2 was sent to parents to complete at home. In total, 4 parents did not complete the CCC-2, of whom 3 were from the VI group.

All stories were pre-recorded such that prosody and speed of speech were alike for each participant. Non-literal utterances were uttered in an appropriate voice, e.g. ironic remarks had a typically ironic voice. The participants first received the non-literal stories, and after that the theory of mind tasks were administered. All stories, including the first- and second order theory of mind stories, were auditorily presented from a laptop. The stories were presented in two different orders. If children provided no answer or an inappropriate answer, a prompt question was given to elicit a response.

For consistency among scoring, an answer protocol was made in advance with possible answers and their corresponding credits. Two persons administered the tests. All responses were rated by them together. To examine the inter-rater reliability, another person not involved in the experiment, acted as second rater. The inter-rater reliability was *k* = 0.80 (*p* < .001) for the non-literal stories and *k* = 0.89 (*p* < .001) for the theory of mind tasks.

## Data Analysis

Participants were divided in a group of younger children (6–9 years, *n* = 26), and older children (10–13 years, *n* = 22). For each participant, a sum score was calculated for the non-literal stories and for the different theory of mind tasks (ToM). Because a child could receive 6 points for the second order tasks, but 4 points for the other theory of mind tasks, the sum scores for the unexpected content tasks and first order theory of mind tasks were weighted by multiplying with a factor of 1.5.

The sum score for the non-literal stories was entered into an ANOVA with Group (control, VI) and Age (young, old) as between-subject factors. The sum scores for the theory of mind tasks were entered into a MANOVA with ToM (1^st^ order, 2^nd^ order) as within-subject factor and Group (control, VI) and Age (young, old) as between-subject factors. For the control story, a non-parametric Mann–Whitney test was used, because these data strongly deviated from a normal distribution. A Pearson’s correlation was calculated between the experimental tasks to check whether these tasks are associated with each other. Moreover, to find out whether the amount of vision played a mediating role, we computed correlations between the experimental tasks and visual acuity. Finally, the composite scores on the CCC-2 were analyzed by computing ANOVA’s, and the standard scores on the subscales were analyzed by a MANOVA. For all comparisons, *p* values smaller than 0.05 were considered significant, and *p* values larger than 0.10 were reported as non-significant (abbreviated to *n.s.*).

## Results

### Experimental Tasks

Tables 2 and 3 show the mean scores on the theory of mind tasks and the non-literal stories.

**Table 2:** Mean scores for the visually impaired group and the control group

Tasks	Mean (SD)	
	VI group	Control group
Unexpected content ToM (weighted)	5.5 (1.1)	6 (0)
1st order ToM (weighted)	4.4 (1.7)	4.2 (2.0)
2nd order ToM	3.1 (2.1)	2.0 (1.8)
Total score ToM (weighted)	13.4 (2.9)	12.1 (3.3)
Total score Non-literal stories	24.0 (3.9)	23.7 (3.6)

**Table 3:** Mean scores for the younger age group and older age group

Task	Mean (SD)	
	Young (6–9 years)	Old (10–13 years)
Unexpected content ToM (weighted)	5.7 (1.0)	5.8 (0.7)
1st order ToM (weighted)	3.5 (1.8)	5.3 (1.3)
2nd order ToM	1.9 (1.9)	3.2 (1.9)
Total score ToM (weighted)	11.2 (3.0)	14.3 (2.5)
Total score Non-literal stories	22.2 (3.5)	25.8 (2.9)

For the unexpected content task, all control children received the maximum score of 4 points, and 2 visually impaired children received 1 or 2 points, and 3 visually impaired children received 3 points (note that in Tables 2, 3, the weighted sum scores are reported). Because of a total lack of variance in the control group, we left the unexpected content task out of statistical analysis.

A MANOVA on first and second order theory of mind scores showed no effect for Group (*F*(2,37) = 1.06, *p* > .10), but there was a significant effect for Age (*F*(2,37) = 6.49, *p* = .004) with an overall better performance for the older children than the younger children, in particular for the first-order false belief task (*F*(1,38) = 12.2, *p* = .001), and a trend for the second-order false belief task (*F*(1,38) = 3.36, *p* = .075). There was no significant interaction of Group by Age (*F*(2,37) < 1, *p* > .10).

An ANOVA on the non-literal stories scores revealed no significant effect for Group (*F*(1,44) < 1, *p* > .10), but there was a significant effect for Age (*F*(1,44) = 14.1, *p* = .001) with a higher performance on non-literal stories for the older children than the younger children. There was no significant interaction of Group by Age (*F*(1,44) < 1, *p* > .10). For the control story involving a causal inference, a non-parametric Mann–Whitney test demonstrated neither an effect for the Group (*U* = 288, *p* > .10) nor for Age (*U* = 282, *p* > .10).

There was a positive relationship between performance on non-literal stories and theory of mind tasks (*r* = 0.58, *p* < .001). However, performance on non-literal stories as well as on theory of mind tasks was not associated with visual acuity in the VI group (both *p* > .10).

### Children’s Communication Checklist (CCC-2 NL)

There was no significant difference between the visually impaired group and control group on the composite score General Communication (*F*(1,42) = 2.39, *p* > .10), but the composite score Pragmatics showed a trend that just failed to reach conventional levels of statistical significance (*F*(1,42) = 3.79, *p* = .058). A MANOVA on the standard scores of the subscales also revealed a trend for Group (*F*(10,33) = 1.89, *p* = .083). The groups differed on the subscales “Non-verbal communication” (*F*(1,42) = 7.63, *p* = .008), and “Inappropriate initiation” (*F*(1,42) = 6.97, *p* = .012), see also Table 4. Note that the mean standard scores across the CCC-2 subscales remained within normal range limits (mean = 10, SD = 3).

**Table 4:** Mean scores on the Dutch version of the CCC-2 composite scores and subscales (standard scores)

CCC-2	Mean (SD)	
	VI group	Control group
General communication score	90.1 (19.4)	81.4 (18.1)
Pragmatics score	47.1 (10.3)	41.2 (9.9)
A. speech	10.0 (3.1)	9.7 (2.9)
B. syntax	10.6 (3.5)	10.0 (3.2)
C. semantics	11.0 (2.7)	10.4 (2.9)
D. coherence	11.3 (3.1)	10.0 (3.1)
E. inappropriate initiation	12.0 (2.8)	9.4 (3.5)
F. stereotyped language	11.2 (3.1)	9.9 (2.4)
G. use of context	11.3 (3.6)	12.0 (3.4)
H. non-verbal communication	12.5 (3.3)	9.9 (3.3)
I. social relations	10.8 (3.3)	10.0 (3.3)
J. interests	11.4 (3.0)	11.0 (2.5)

Note that higher scores indicate more difficulties. For standard scores, mean = 10, SD = 3

## Discussion

This study explored whether children (aged 6–13 years) with varying congenital visual impairments show a delayed development in advanced theory of mind. More specifically, we examined the understanding of first-order and second-order theory of mind as well as non-literal language. Because previous research has found that children with congenital blindness or profound visual impairment are later at developing a theory of mind (Brambring and Asbrock 2010; Green et al. 2004; McAlpine and Moore 1995; Minter et al. 1998; Peterson et al. 2000; Roch-Levecq 2006), we hypothesized that more advanced theory of mind understanding might be delayed as well. However, this hypothesis was not confirmed. When compared with sighted children who were matched on age and verbal intelligence, children with varying congenital visual impairment had a similar performance on advanced theory of mind stories and non-literal stories. In both groups, older children (aged 10–13 years) had a better performance on the theory of mind tasks and non-literal stories than younger children (aged 6–9 years). That is, we did not find evidence for a delay in advanced theory of mind development in visually impaired children. Moreover, within the visually impaired group, the degree of visual acuity loss did not appear to play a role on task performance.

The current findings seem to be in contrast with previous research on theory of mind in children with visual impairments. For instance, McAlpine and Moore (1995) found that the severity of visual impairment affects performance on a false belief task. However, it has to be noted that McAlpine and Moore (1995) used tactile theory of mind tasks only. We argue that the divergent findings might be explained by the kind of task that was used: tactile versus verbal tasks. The current results showed that some visually impaired children had difficulty with the unexpected content task, which was a relatively simple task that should be solved by touch or vision. Whereas all control children received the maximum score, 21% of the children with a visual impairment did not. It is this kind of tactile tasks that is often used to investigate first-order theory of mind in children with visual impairment (Brambring and Asbrock 2010; Green et al. 2004; McAlpine and Moore 1995; Minter et al. 1998; Peterson et al. 2000). Therefore, delays in theory of mind that have been found may be—at least partly—due to task-related factors (Brambring and Asbrock 2010).

Except for the unexpected content task, all tasks included auditorily presented stories. The processing of such stories may place a high demand on working memory capacity. That is, verbal information such as story characters, plot and story events have to be kept in memory until the questions are asked, and participants need to actively use the story information. One possible explanation for the current findings is that visually impaired children are superior in processing auditory information compared to control children. In line with this, it has been found that blind people have a better memory for auditory verbal information (Hull and Mason 1995; Raz et al. 2007; Roder et al. 2001; Swanson and Luxenberg 2009; Edmonds and Pring 2006). Moreover, Tadic et al. (2010) showed that children with visual impairment were superior at recalling sentences, which also points to a verbal memory advantage.

Our finding that children with visual impairment performed similar on non-literal language comprehension as sighted children, seems to be at odds with Pring et al. (1998). They found that visually impaired children identified less accurately the motivation behind a character’s non-literal utterance in everyday stories (Pring et al. 1998). However, this discrepancy among findings may have two reasons. First, Pring et al. (1998) included only blind or profoundly visually impaired children. Second, they included more different story types, including persuasion, misunderstanding, contrary emotions, and double bluff. Such non-literal language is likely to be more difficult than most story types used in our study (O’Hare et al. 2009). Therefore, one should be cautious when comparing findings.

The current results indicate that children with congenital visual impairment—despite a limited access to visual information during interactions (e.g. joint attention, mutual gaze, facial expressions, and gestures)—can develop an effective theory of mind. However, these findings do not imply that those children do not experience any difficulties with pragmatics. Theory of mind and non-literal language involve only a small portion of pragmatics. Our evaluations of language and communication skills by means of the CCC-2 at least suggests that visually impaired children may have problems with other aspects of pragmatics, in particular, initialization of conversation and non-verbal communication. These aspects of pragmatics perhaps rely more directly on visual information such as eye contact and facial expressions than non-literal language. Such aspects of pragmatics would provide a direction for further research.

A limitation of this study is the relatively large group of older children, in particular for the most severely visually impaired children. Therefore, it is possible that we could have missed an early delay in theory of mind development. Still, it is remarkable that even with limited access to visual information during interactions, like joint attention, mutual gaze, face expressions, and gestures, children with congenital visual impairment can develop an effective theory of mind. In line with our findings, it has been found that congenitally blind adults acquire a typical theory of mind brain network, despite absence of visual experience during development (Bedny et al. 2009). One way to compensate for a limited access to visual information during interaction might be a stronger reliance on language (Bedny et al. 2009; Brambring and Asbrock 2010). It is therefore conceivable, that the visually impaired children in the present study have developed an effective theory of mind by using language as a major source of information to compensate for a lack of vision. Future research needs to be conducted to test this claim. Finally, because of large heterogeneity in etiology within the group of visually impaired children, longitudinal research may give more insight into the individual trajectories in social-cognitive development.

## Conclusion

The present findings showed that, when using appropriate, verbal tasks, children with a variety of congenital visual impairments had no developmental delay in more advanced theory of mind understanding. Despite a limited access to visual information during interactions (e.g. joint attention, mutual gaze, facial expressions, and gestures), children with congenital visual impairment can develop an effective theory of mind.

## Appendix A

See Table 5.

**Table 5:** Clinical characteristics of individual children with visual impairment

No	Age (years)	Gender	Visual acuity^a^	Visual impairment
1	6.6	m	0.05	Albinism
2	7.1	f	0.05	Iris coloboma; hydrocephalus; microphthalmus
3	7.3	f	0.12	Albinism
4	7.4	f	0.25	Aniridia; nystagmus
5	8.2	f	0.16	Achromatopsia
6	8.5	m	0.125	High hypermetropia
7	8.5	m	0.25	Congenital cataract, amblyopia
8	8.8	m	0.08	Retinal scarring after herpes infection
9	8.8	m	0.06	Nystagmus
10	9.6	f	0.3	Albinism
11	10.1	m	0.04	Persistent hyperplastic primary vitreous (PHPV)
12	10.8	m	0.18	Albinism
13	10.8	f	0.05	Tapetoretinal dystrophy (TRD)
14	11.0	f	0.25	Albinism; nystagmus
15	11.3	f	0.16	Congenital cataract
16	11.4	m	0.12	Macular hypoplasia
17	11.5	f	0.05	Retinopathy of prematurity (ROP)
18	11.7	m	0.1	Nystagmus, achromatopsia
19	11.7	f	0.12	Microphtalmus
20	11.8	m	0	Crouzon syndrome
21	11.9	f	0	Tapetoretinal dystrophy (TRD)
22	12.3	m	0	Leber’s congenital amaurosis (LCA)
23	13.2	f	0.05	Retinoblastoma
24	13.2	f	0.08	Nervus opticus atrophy

^a^Visual acuity of (corrected) best eye in Snellen decimals

## Appendix B

See Table 6.

**Table 6:** Theory of mind stories

Theory of mind	Stories (translated from Dutch)	Questions
1st order false belief	Tom and Lotte are playing in the class room. Tim needs to go to the toilet and is leaving the class room. Lotte does not like to play on her own and is going to the kitchen to get a glass of lemonade. When Tim comes back from the toilet, he wants to play again with Lotte.	(1) Where is Tom going to play again with Lotte? (2) Why does Tim go there? *Control questions:* Where did he see Lotte the last time? Where is Lotte now?
	Bob and Roos are playing upstairs. Bob is playing with his favorite marble. Then the telephone is ringing downstairs. He puts his marble in his marbles pouch onto the floor. When he is downstairs, Roos sneaks Bob’s favorite marble out of his marbles pouch and hides the marble in her trouser pocket. After a few minutes, Bob returns and he wants to play again with his favorite marble.	(1) Where will Bob look first for his marble? (2) Why will Bob look first there? *Control questions:* Where did Bob put the marble when we went downstairs? Where is the marble now?
2nd order false belief	Janneke en Marloes are playing in Marloes’ room. Marloes has a letter from her friend Bram. Janneke really wants to know what is written in the letter but Marloes does not want her to read it. Marloes’ mom calls her. She puts the letter under the blankets of her bed, and leaves the room. While Marloes is gone, Janneke takes the letter and reads it sneakily. Then she puts it away in Marloes’ desk. When Marloes comes back, she sees Janneke putting the letter in the desk, but Janneke does not notice that Marloes has seen her putting the letter in the desk. *…* Later on, Marloes says to Janneke, ‘‘OK, you may the read letter.’’ And she goes to get the letter.	(1) Where does Janneke think Marloes will look for her letter? (2) Why does Janneke think this? *Control questions:* Does Janneke know that Marloes saw her? Where did Marloes put her letter before she went to see her mom? Where is the letter now? Does Marloes know where the letter is now?
	Joris and Inge are in the park. In the park there is an ice-cream van. Joris would like to buy an ice-cream for both, but he has left his money at home. The ice-cream man says: “You can fetch your money at home. I’ll be here in the park all afternoon.”Joris goes home to fetch his money. Meanwhile, Inge is waiting for him at the ice-cream van. The ice-cream man changed his mind en says to Inge: “I will no longer stay in the park, I am going to drive my van to the market square.” The ice-cream man drives over to the market square. On his way, he meets Joris and tells him that he’s going to drive his van to the market square. Meanwhile, Inge is going to Joris’ home to pick him up. When she arrives at his home, his mother tells her he is gone to buy an ice-cream.	(1) Where does Inge think Joris is going to buy an ice-cream? (2) Why does she think he has gone there? *Control questions:* Does Inge know that Joris met the ice-cream man? Does Joris know where the ice-cream man is now? Where is he now?

## Appendix C

See Table 7.

**Table 7:** Non-literal language stories

Type of story	Example (translated from Dutch)
Lie	Daan is playing in the living room. He accidentally knocks over his mother’s favorite vase. The vase falls down on the floor and breaks into a lot of pieces. Oh dear, when mother finds out she will be very cross, he thinks. When Daan’s mother comes home, she immediately sees the broken vase. She asks Daan: what happened with my vase. Daan says, “The dog knocked it over, it wasn’t my fault!”
	Pieter hates eating boiled sprouts. His mother tells him that they will eat boiled sprouts tonight. Pieters tells his mother: “I am having a terrible bellyache.”
White lie	Maaike asked her favorite aunt a pet for her birthday present. Maaike really loves having a rabbit and she hopes that her aunt will give her a rabbit. Today it is her birthday. Her aunt has a big box with her and Maaike feels sure it will contain a rabbit. But when she opens it, she finds a fish bowl with a goldfish. Maaike thinks a goldfish is very boring. When her aunt asks her: and, do you like your present?, Maaike says: “What a lovely present”.
	Marieke baked an apple pie today. She has been in the kitchen the whole afternoon to make the pie. Marieke gives Lars a piece of her pie. Lars takes a big bite. He finds it disgusting. When Marieke asks: “Do you like my apple pie?”, Lars says: “Oh, it is tasty”.
Figure of speech	In Joost’ class, there is a new girl. He really likes the girl. He is trying to come near her all the day, and he is very happy. When his mother asks him why he is so happy, he says: “I am having butterflies in my stomach”.
	Mark just woke up and is having breakfast. He does not like to go to school today and is cross when his mother asks him what kind of sandwich filling he likes to have. His mother says: “Oh, you must have gone out of bed on the wrong side”.
Joke	Today Jeroen is going to Emma’s house for the first time to play with her. Emma told him she is having a dog. Jeroen likes dogs very much and he is looking forward to seeing her dog. When Jeroen arrives at Emma’s house Emma’s runs to open the door, and her dog jumps up to greet Jeroen. Emma’s dog is huge, it’s almost as big as Jeroen! When Jeroen sees Emma’s huge dog, he says: “Emma, you haven’t got a dog at all. You’ve got an elephant!”
	Today Annelies is going to visit Thomas. Thomas told her that his cat has a nest with little kittens. His cat is not an ordinary cat, but a purebred cat with a lot of hair. The kittens also have a lot of hair already. When Annelies arrived, she immediately looks at the kittens. When Annelies sees the kittens, she says: “Thomas you haven’t got kittens at all. You’ve got a lot of balls of wool!”
Irony	Today Sarah and Tom are going to the beach for swimming and sunbathing. It is Tom’s idea, he says it is going to be a lovely sunny day for going to the beach. But just as they are arriving at the beach, it starts to rain and thunder. Sarah is cross. She says ‘‘Oh yes, a lovely day for going to the beach”.
	It is holiday. Stefan and Kim are sitting in the backseat of the car. They are quarreling all the time. Their father says: “Oh, it is so pleasant again”.
Control (causal inference)	Laura is in the garden. She is sowing seeds, so that next year she will have lots of vegetables in her garden. She sows seeds for cauliflower and carrots. She sows the seeds well, but when she goes inside after sowing them, the birds fly down and eat up all her seeds! (1) Did Laura sow seeds? (2) Why will Laura not have any vegetables in her garden next year?
